# Dataset from the zero-energy log house project

**DOI:** 10.1016/j.dib.2020.106509

**Published:** 2020-11-07

**Authors:** Antti Kosonen, Anna Keskisaari

**Affiliations:** LUT School of Energy Systems, Lappeenranta-Lahti University of Technology LUT, Lappeenranta, 53850 Finland

**Keywords:** Zero energy, Solar PV, Heat pump, Log house, Energy efficiency

## Abstract

In this data article, we present a supplementary dataset from a zero-energy log house project in southern Finland, presented in detail in [1]. This article consists of comprehensive energy-related data collected in practice from several sources from the house during the period of 2017−2019. The data include solar PV production data of two separate systems, in south and east−west directions. The solar PV data are presented on a different time scale to demonstrate the operation of two installations in different seasonal conditions. Simulated results are also included. The electrical energy consumption is distributed between the consumer, ventilation, the ground source heat pump for space heating and the domestic hot water energy at the monthly level. The realized electrical energy prices, self-sufficiency rates, and costs are also presented at the monthly level. The heat production of the ground source heat pump is estimated according to the service hour data and the performance data given by the manufacturer. The data can be applied in new and building-under-renovation projects.

## Specifications Table

**Table:** 

Subject	Renewable Energy, Sustainability and the Environment
Specific subject area	Building engineering, Zero-energy building, Energy efficiency
Type of data	Table, image, figure
How data were acquired	Data reading from the devices, notes, SPOT electricity market price data Instruments / software: Automatic meter reading: Aidon 6460SE MeshNET / local electricity transmission company online web system Ventilation unit: Enervent Pandion MDE-CHG / Enervent eAir web Solar PV inverter: SMA Sunny Tripower 7000TL and 9000TL / Sunny Explorer / Rasperry PI-based software Solar PV simulation: HOMER software Heat pump: Nibe F1145-6 / NIBE Uplink^TM^ Power monitoring: Siemens SENTRON PAC3200 / Rasperry PI-based software
Data format	Raw, analyzed, filtered
Parameters for data collection	The data have been collected from three years of practical use of the house during the period of 2017−2019. During that period, two adults were living in the house.
Description of data collection	The data have been collected from the devices used in the house by the software offered by the manufacturers and Rasperry PI-based software.
Data source location	Building: Zero-Energy Log House City: Imatra Country: Finland Latitude and longitude for the collected samples/data: 61°08’46’’N 28°46’13’’E
Data accessibility	With the article
Related research article	A. Kosonen, A. Keskisaari, Zero-energy log house – Future concept for an energy efficient building in the Nordic conditions, Energ. Buildings 228 (2020). https://doi.org/10.1016/j.enbuild.2020.110449.

## Value of the Data

• Measured three-year operational energy data in a single-family log house in southern Finland that is a plus energy building. Energy data consist of south and east–west facing solar PV system productions in the same site, a ground source heat pump (GSHP) as a main heat source, other electricity consumption (ventilation and consumer), self-sufficiency rates of the solar PV electricity without a stationary battery when the GSHP-based heating is controlled.
• The data can be valuable and useful for researchers, engineers, architects, manufacturers, designers, energy policy makers, and end-users. In addition, data work as an eye-opener for them who are involved in the future plus energy buildings.
• The measured practical dataset can be used as a reference material to verify models, load and production forecasting, and practical data in other similar buildings. In addition, it works as an education material.

## 1 Data Description

**Fig. 1:**
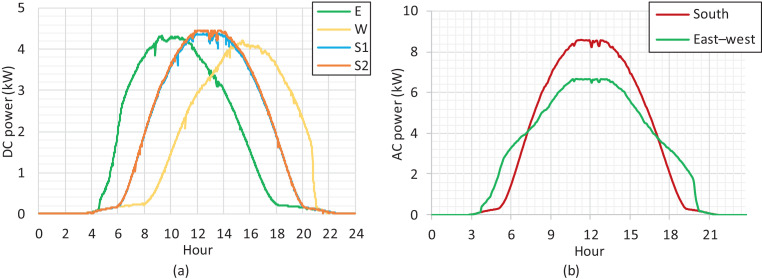
Solar PV production on a clear summer day on 15 June 2017. (a) Tracker-specific production on the DC side. (b) Inverter-specific production on the AC side.

**Fig. 2:**
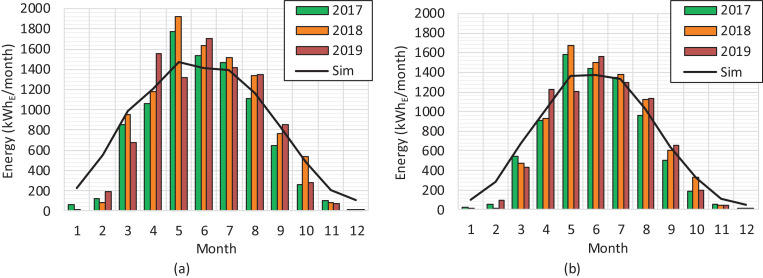
Solar PV electricity at the monthly level during the period of 2017−2019. (a) Total production of the south system. (b) Total production of the east–west system.

A shadowless location guarantees high performance of the solar PV plant production at an annual level to reach the local production potential estimation, which is about 1000 kWh_E_/m^2^ in southern Finland [2]. The position of the sun varies considerably in Nordic locations depending on the season, from a low position in wintertime with a short day length to a high position in summertime with a long day length. The production curves for each panel string (east 5.355 kWp, west 5.355 kWp, south1 5.200 kWp, south2 5.200 kWp) on a clear summer day are presented in Fig. 1(a). The inverter-specific production curves (south 10.4 kWp, east–west 10.71 kWp) for the same day are presented in Fig. 1(b). All the raw data for the figures are reported in the Supplementary Material. The monthly level productions both for the south and east–west installations during a three-year period including local simulated production are presented in Fig. 2.

**Fig. 3:**
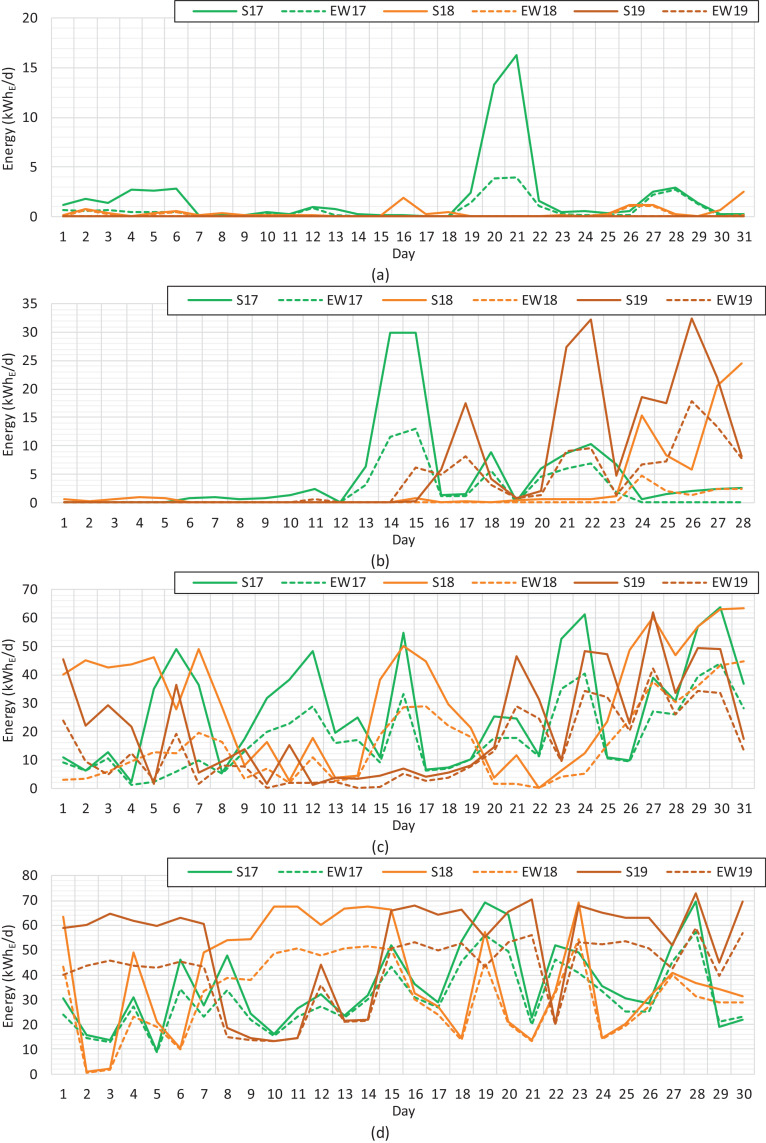
Solar PV electricity at the daily level during the period of 2017−2019. (a) January. (b) February. (c) March. (d) April.

**Fig. 4:**
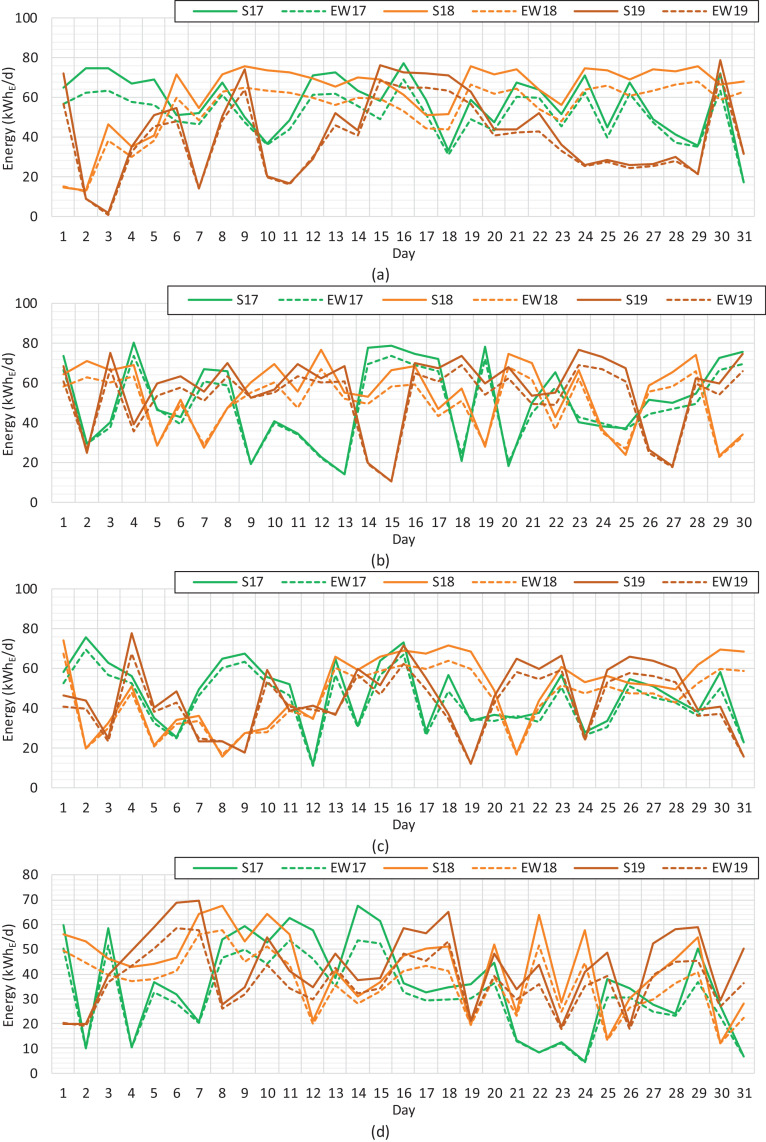
Solar PV electricity at the daily level during the period of 2017−2019. (a) May. (b) June. (c) July. (d) August.

**Fig. 5:**
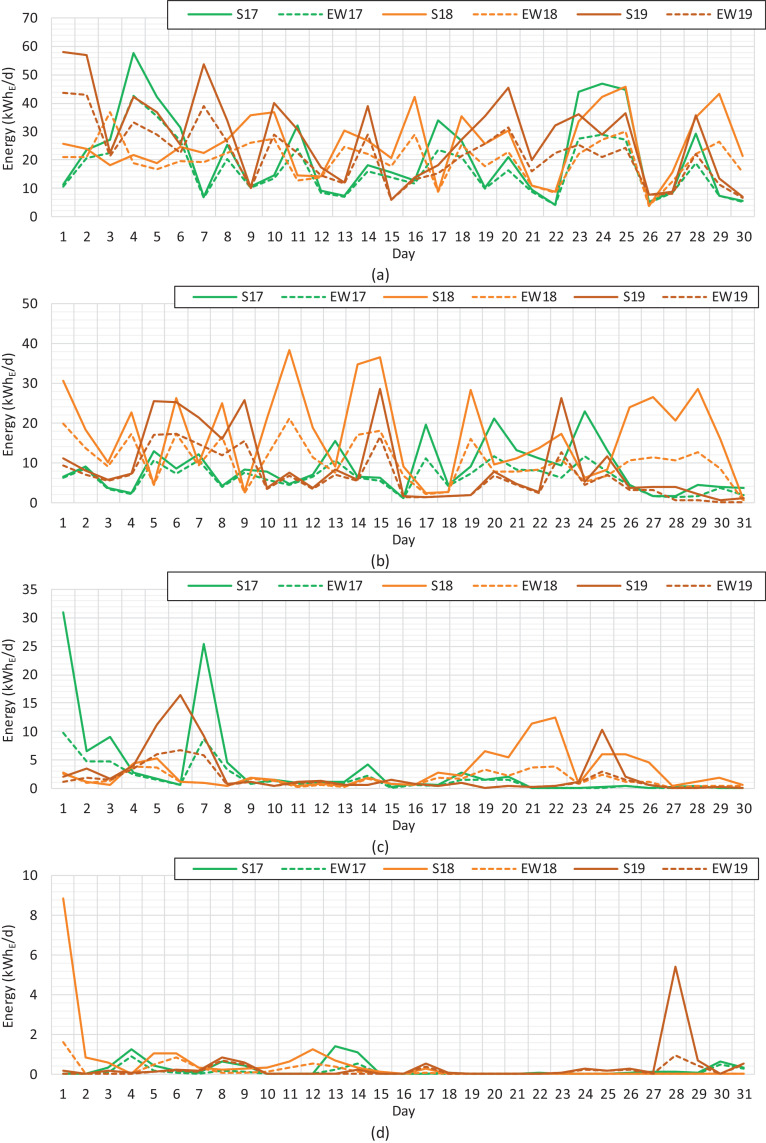
Solar PV electricity at the daily level during the period of 2017−2019. (a) September. (b) October. (c) November. (d) December.

The performance of the solar PV production is generally described with annual production related to the installed capacity, generally called specific yield. The specific yields of solar PV electricity for the south and east–west installations are gathered in Table 1. The seasonal performance of the solar PV installations with unequal orientations can be seen in the daily graphs. Fig. 3, Fig. 4, Fig. 5 show the daily solar PV production rates of the south and east–west installations for each month during a three-year period.

**Table 1:** Specific yields of solar PV electricity at the monthly level during the period of 2017−2019.

	Specific yield (kWh_E_/kWp)												
Year	*Jan*	*Feb*	*Mar*	*Apr*	*May*	*Jun*	*Jul*	*Aug*	*Sep*	*Oct*	*Nov*	*Dec*	*Total*
	South												
Sim.	22.06	53.21	94.86	115.77	141.42	135.92	134.07	111.69	80.42	46.57	20.18	10.05	966.22
2017	5.60	12.10	81.80	102.03	170.89	147.63	140.72	107.00	61.72	25.06	9.65	0.69	864.88
2018	1.12	7.93	91.99	113.33	185.13	157.11	146.16	129.16	73.41	51.29	8.26	1.61	966.50
2019	0.00	18.64	64.60	149.27	127.19	163.89	135.91	129.57	81.81	27.31	7.02	1.02	906.23

	East–west												
Sim.	8.88	26.48	62.41	94.55	126.86	128.51	124.22	96.24	59.68	30.21	10.07	4.39	772.49
2017	2.01	5.16	50.38	84.85	147.63	134.06	125.76	89.27	46.78	17.83	4.61	0.27	708.61
2018	0.38	1.19	44.09	86.95	156.57	139.87	128.57	105.35	56.17	30.39	4.23	0.46	754.22
2019	0.00	9.11	39.93	114.40	112.27	146.18	121.26	106.04	60.77	18.57	3.92	0.42	732.86

**Fig. 6:**
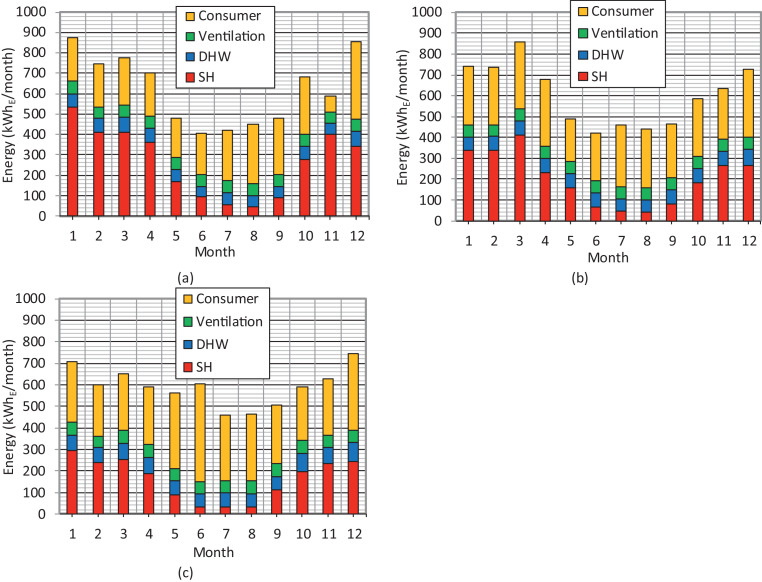
Distribution of the electricity consumption at the monthly level during the period of 2017−2019. The space heating is marked as SH and the domestic hot water as DHW. (a) 2017. (b) 2018. (c) 2019.

The distribution of electricity consumption at the monthly level in the zero-energy log house for a three-year period is illustrated in Fig. 6. The solar PV electricity self-sufficiency rates of the consumption according to [3] at the monthly level are depicted in Table 2. According to [4], the purchased electricity price is approximately three times as high as the price of the sold electrical energy. The realized purchased and sold electricity prices without basic fee at the monthly level during a three-year period are gathered in Table 3. The realized electricity total cost for the purchased electricity with a monthly basic fee and the sold and self-consumption electrical energy at the monthly level during a three-year period are gathered in Table 4.

**Table 2:** Electricity self-sufficiency rates of the consumption at the monthly level during the period of 2017−2019.

	Self-sufficiency rate (%)												
Year	*Jan*	*Feb*	*Mar*	*Apr*	*May*	*Jun*	*Jul*	*Aug*	*Sep*	*Oct*	*Nov*	*Dec*	*Avg.*
2017	5.10	8.74	38.55	57.36	80.43	80.67	75.18	58.72	48.20	30.50	10.97	1.06	35.04
2018	1.63	6.79	45.13	60.21	69.73	74.21	69.86	60.13	55.10	43.08	11.06	1.84	36.87
2019	0.00	15.62	41.99	67.85	68.88	53.34	72.76	66.73	55.03	31.12	9.36	1.66	36.97

**Table 3:** Realized electricity prices without basic fee at the monthly level during the period of 2017−2019.

	Electricity price (c/kWh_E_)												
Year	*Jan*	*Feb*	*Mar*	*Apr*	*May*	*Jun*	*Jul*	*Aug*	*Sep*	*Oct*	*Nov*	*Dec*	*Avg.*
	Purchase												
2017	9.87	10.09	9.46	9.41	9.32	9.02	9.74	9.87	10.44	9.72	9.88	9.62	9.78
2018	10.71	11.37	11.39	10.85	9.92	11.51	12.50	12.60	12.01	11.85	12.29	12.60	11.64
2019	13.63	12.56	11.64	11.71	10.89	9.75	11.77	11.98	12.14	12.25	12.37	11.39	12.07

	Sold												
2017	2.94	3.55	3.13	3.30	3.08	3.46	3.45	3.88	4.01	3.86	3.06	3.32	3.43
2018	3.99	7.25	4.88	4.06	4.76	4.85	5.50	5.75	5.47	4.73	5.12	4.70	5.03
2019	-	4.18	3.73	4.23	4.50	3.70	5.03	5.43	5.48	5.18	5.03	3.26	4.57

**Table 4:** Realized electricity total costs at the monthly level during the period of 2017−2019.

	Electricity cost (eur/month)												
Year	*Jan*	*Feb*	*Mar*	*Apr*	*May*	*Jun*	*Jul*	*Aug*	*Sep*	*Oct*	*Nov*	*Dec*	*Total*
	Purchase with fixed cost												
2017	97.1	83.7	60.2	43.3	23.9	22.3	25.3	33.4	41.1	61.3	67.1	96.7	655.2
2018	94.9	94.5	70.1	45.6	31.0	28.1	33.7	38.1	41.0	55.8	86.0	106.5	725.3
2019	115.7	82.6	63.2	41.1	37.8	46.0	33.3	36.9	46.1	69.1	89.7	103.0	764.5

	Sold												
2017	−1.0	−4.1	−34.1	−51.8	−91.6	−91.5	−86.1	−70.1	−36.6	−9.4	−2.6	0.0	−479.0
2018	−0.1	−3.3	−50.8	−69.2	−155.4	−137.7	−141.7	−127.3	−60.9	−28.7	−3.1	−0.4	−778.7
2019	0.0	−8.3	−30.9	−100.8	−96.7	−109.3	−120.4	−118.8	−67.5	−15.5	−2.8	−0.1	−671.0

	Total												
2017	96.1	79.6	26.0	−8.5	−67.7	−69.2	−60.9	−36.7	4.5	51.9	64.5	96.7	176.3
2018	94.7	91.3	19.3	−23.6	−124.3	−109.6	−107.9	−89.2	−19.9	27.0	82.9	106.1	−53.4
2019	115.7	74.3	32.3	−59.7	−58.9	−63.2	−87.1	−81.9	−21.4	53.6	86.9	102.9	93.4

	Self-consumption												
2017	−4.5	−6.8	−29.4	−39.9	−37.7	−32.7	−32.8	−28.0	−24.9	−21.9	−6.4	−0.9	−265.9
2018	−1.3	−6.8	−46.2	−45.4	−36.6	−37.1	−41.9	−35.3	−32.7	−31.2	−8.7	−1.7	−324.9
2019	0.0	−11.4	−31.8	−48.9	−43.5	−36.1	−43.5	−41.9	−38.1	−24.5	−7.4	−1.4	−328.5

**Fig. 7:**
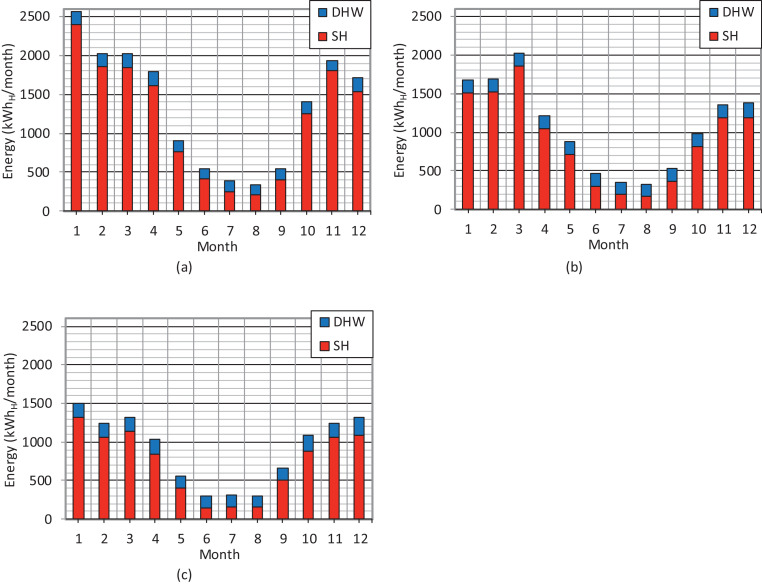
Distribution of the estimated heating energy produced by the ground source heat pump at the monthly level during the period of 2017−2019. (a) 2017. (b) 2018. (c) 2019.

In the zero-energy log house, the main heating system is based on a ground source heat pump. The service hours at the monthly level both for the space heating (SH) and the domestic hot water (DHW) during a three-year period are presented in Table 5. The distribution of the estimated heating energy produced by the heat pump at the monthly level during the three-year period is illustrated in Fig. 7.

**Table 5:** Service hours of the ground source heat pump at the monthly level during the period of 2017−2019.

	Service hours (h)												
Year	*Jan*	*Feb*	*Mar*	*Apr*	*May*	*Jun*	*Jul*	*Aug*	*Sep*	*Oct*	*Nov*	*Dec*	*Total*
	Space heating (SH)												
2017	355	275	273	239	112	62	36	30	60	185	267	227	2121
2018	224	225	275	155	105	45	30	26	54	120	176	176	1611
2019	196	158	168	125	59	22	23	23	75	130	157	162	1298

	Domestic hot water (DHW)												
2017	45	45	50	49	40	34	39	37	37	42	35	49	502
2018	43	45	45	45	45	45	40	40	46	47	46	51	538
2019	48	47	51	51	43	41	41	39	42	57	49	59	568

## 2 Experimental Design, Materials and Methods

**Fig. 8:**
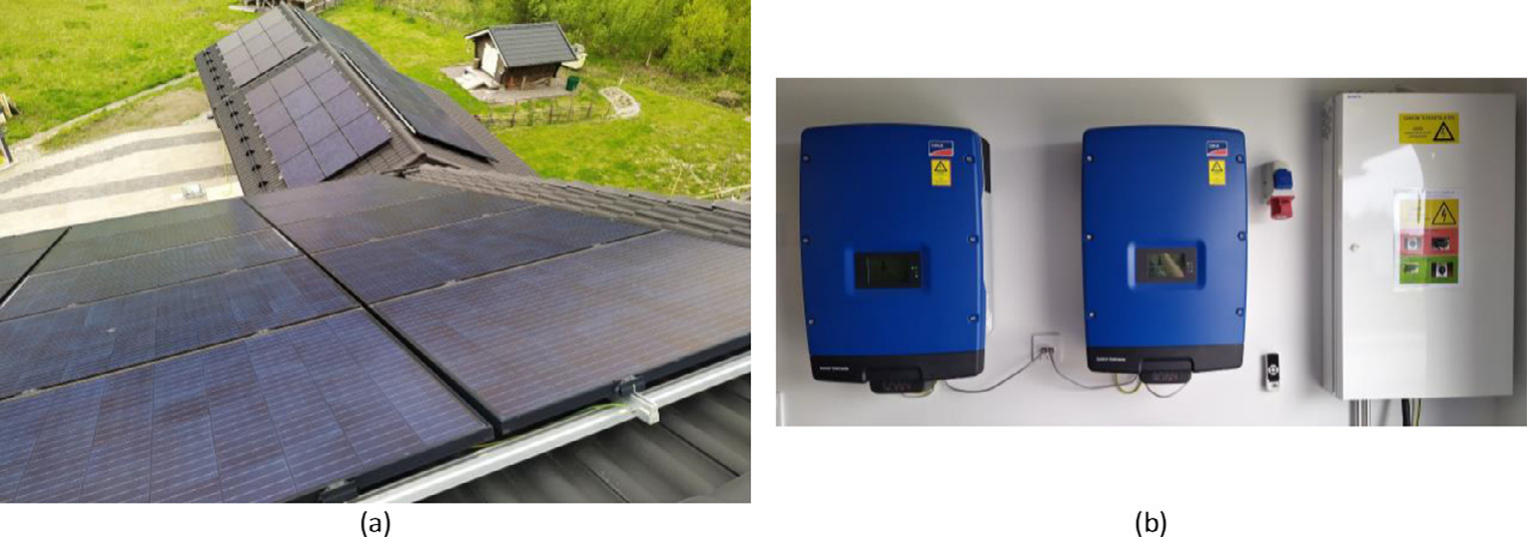
Solar PV system installations. (a) Panels. (b) String inverters.

The solar PV production simulations were driven by the HOMER software, which uses weather and irradiation data gathered by NASA [5]. The solar PV production data with 5 min resolution were collected monthly from the interface of the SMA Sunny Tripower inverters (7000TL for the east–west system and 9000TL for the south system, see Fig. 8) by using the Sunny Explorer software. In addition, DC and AC power data were collected from the inverters with the Rasperry PI-based software through the SunSpec® Modbus® interface in a 5 s time frame. The solar PV system installations are presented in Fig. 8.

**Fig. 9:**
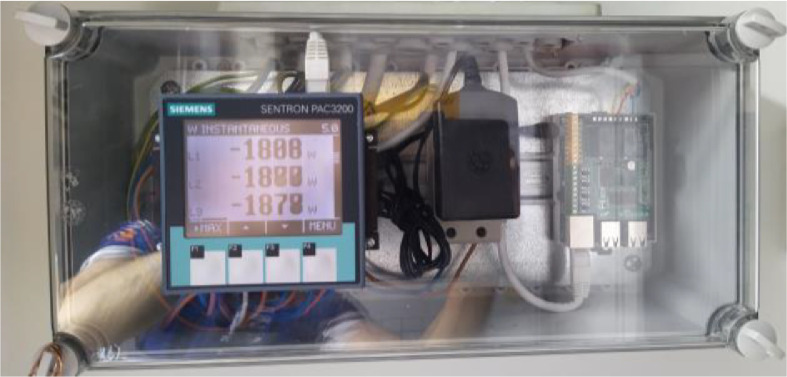
Power monitoring and Rasperry PI hardware.

The purchased and sold electrical energy values were collected through an online web system of the local electricity distribution company at an hourly resolution. The automatic meter reading device was Aidon 6460SE MeshNET, which measures power instantly from each three phases separately and records the measured electrical energy to two registers (purchase and sale). Both the purchase and sale can occur at the same time, if there is an imbalance between production and consumption. In addition, electrical power/energy data were collected from a Siemens SENTRON PAC3200 power monitoring device with the Rasperry PI-based software through the Modbus® TCP interface in a 5 s time frame. The power monitoring and Rasperry PI hardware installations are presented in Fig. 9. Hourly electrical energy prices were collected from the historical market data service of Nord Pool [6].

The service hour values of the Nibe F1145-6 ground source heat pump were collected through the NIBE Uplink^TM^ online web system. The measured input power of the heat pump was about 1.5 kW. Based on the manufacturers’ information, the COP values used for the space and DHW heating were 4.5 and 2.5, respectively. The measured input power of the ventilation machine was about 80 W.

Data were handled and plotted mainly by the Excel software, but also MATLAB was used. Electrical energy self-consumption was calculated as follows

(1) $$E_{E,\text{self}-\text{consump}.}=E_{E,\;\text{solarPV}}-E_{E,\;\text{sold}}$$

where $E_{E,\;\text{solarPV}}$ is the electrical energy produced by the solar PV systems, and $E_{E,\;\text{sold}}$ is the electrical energy sold to the grid. The consumption was calculated as follows:

(2) $$E_{E,\text{consump}.}=E_{E,\text{self}-\text{consump}.}+E_{E,\text{purchased}}$$

where $E_{E,\;\text{purchased}}$ is the electrical energy purchased from the grid.

## CRediT Author Statement

Antti Kosonen: Conceptualization, Methodology, Software, Validation, Formal analysis, Investigation, Resources, Data curation, Writing - original draft, Writing - review & editing, Visualization.

Anna Keskisaari: Conceptualization, Methodology, Validation, Investigation, Resources, Writing - original draft, Writing – review & editing.

## Declaration of Competing Interest

The authors declare that they have no known competing financial interests or personal relationships that could have appeared to influence the work reported in this paper.

## Appendix Supplementary materials

Image, application 1
